# A qPCR-Based Survey of *Haplosporidium nelsoni* and *Perkinsus* spp. in the Eastern Oyster, *Crassostrea virginica* in Maine, USA

**DOI:** 10.3390/pathogens9040256

**Published:** 2020-03-31

**Authors:** Nicholas D. Marquis, Theodore J. Bishop, Nicholas R. Record, Peter D. Countway, José A. Fernández Robledo

**Affiliations:** 1 Bigelow Laboratory for Ocean Sciences, Boothbay, ME 04544, USA; nmarquis1980@gmail.com (N.D.M.); theodorejbishop@smccme.edu (T.J.B.); nrecord@bigelow.org (N.R.R.); pcountway@bigelow.org (P.D.C.); 2 Department of Marine Sciences, Southern Maine Community College, South Portland, ME 04106, USA

**Keywords:** Alveolate, Ascetospora, bivalves, host-parasite interaction, parasite association

## Abstract

Eastern oyster (*Crassostrea virginica*) aquaculture is increasingly playing a significant role in the state of Maine’s (USA) coastal economy. Here, we conducted a qPCR-based survey for *Haplosporidium nelsoni*, *Perkinsus marinus*, and *Perkinsus chesapeaki* in *C. virginica* (n = 1440) from six Maine sites during the summer–fall of 2016 and 2017. In the absence of reported die-offs, our results indicated the continued presence of the three protozoan parasites in the six sites. The highest *H. nelsoni* qPCR-prevalence corresponded to Jack’s Point and Prentiss Island ($\bar{x}=$ 40 and 48% respectively), both located in the Damariscotta River Estuary. Jack’s Point, Prentiss Island, New Meadows River, and Weskeag River recorded the highest qPCR-prevalence for *P. marinus* (32–39%). While the *P. marinus* qPCR-prevalence differed slightly for the years 2016 and 2017, *P. chesapeaki* qPCR-prevalence in 2016 was markedly lower than 2017 (<20% at all sites versus >60% at all sites for each of the years, respectively). Mean qPCR-prevalence values for *P. chesapeaki* over the two-year study were ≥40% for samples from Jack’s Point (49%), Prentiss Island (44%), and New Meadows River (40%). This study highlights that large and sustained surveys for parasitic diseases are fundamental for decision making toward the management of the shellfish aquaculture industry, especially for having a baseline in the case that die-offs occur.

## 1. Introduction

**Figure 1:**
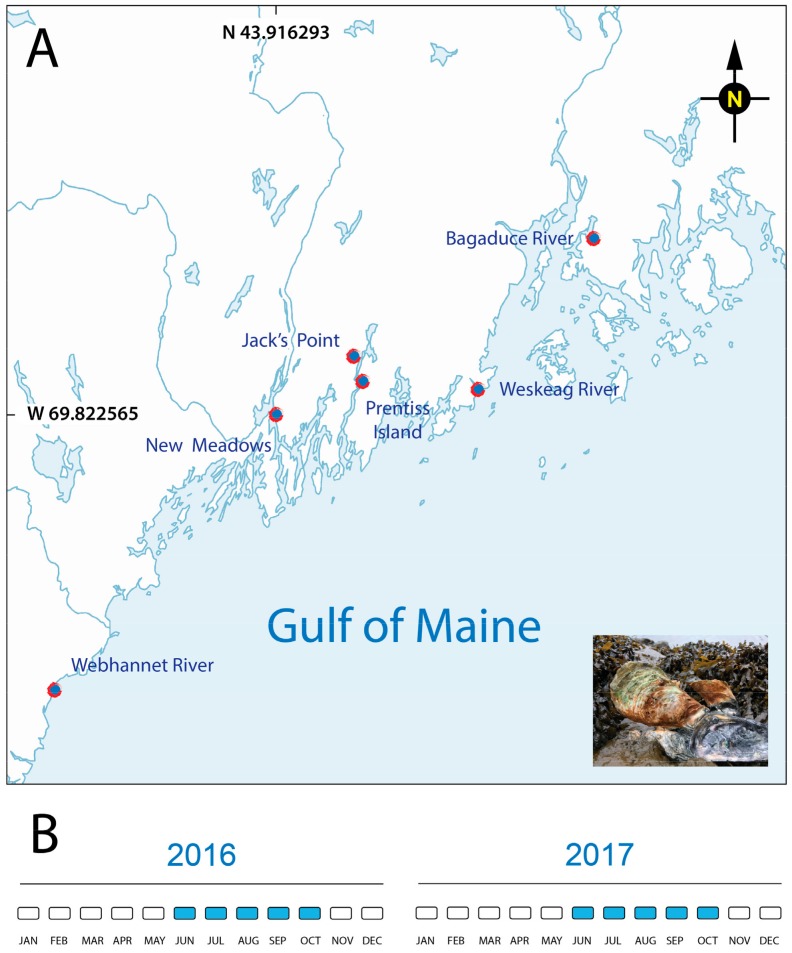
Oyster collection sites in the Gulf of Maine (USA) tributaries (red marks). (**A**) Adult eastern oysters (*Crassostrea virginica*, inset photograph) were collected from shellfish farmers located at six oyster leases along the coast of the Gulf of Maine. (**B**) Sampling calendar for both years of the project.

Marine mollusks are prone to epizootic episodes of a different nature (reviewed in [1]), with new reports of mortalities, as more countries move from fisheries to aquaculture practices and monitoring systems are in place [2,3,4,5]. The eastern oyster is an indigenous species to Maine [6], with oyster aquaculture leases in state waters first granted in 1975 (University of Maine Aquaculture Research Institute, 2014). However, the expansion of the industry has accelerated rapidly over the past two decades, bolstered by the popularity of raw oyster bars. Miles of shoreline in Maine and historically low occurrence of bivalve die-offs make oyster aquaculture a profitable business, despite the cold winters. In other locations, the expansion of shellfish aquaculture has been accompanied by many biological threats in the form of protozoan parasites, bacteria, and other disease-causing agents. Although the literature is sparse, Maine is not immune to these threats [7]. This is particularly true for MSX (multinucleated sphere unknown, now known as *Haplosporidium nelsoni*) and Dermo (originally *Perkinsus marinus*, now also *Perkinsus chesapeaki*) [8,9,10,11,12]. The two major eastern oyster die-offs reported in Maine (1990s and 2010) were both attributed to *H. nelsoni* [13,14]. A survey conducted one year after the 2010 die-off was negative for MSX in Maine oysters [15]. This result, along with the absence of oyster die-offs and the use of MSX-resistant oyster strains [16], suggests that MSX is under control [17]. Interestingly, switching from an MSX susceptible to MSX resistant oyster strain has not eliminated the parasite from Maine oysters. Instead, MSX continues to be present in Maine, as revealed by several PCR-based studies published by different research groups [9,10,11,12,18]. The presence of *P. marinus* was first reported in Maine at “sub-clinical” levels in 1995 [19]. The PCR-prevalence (percentage of oysters positive for the parasite) for both *P. marinus* and *P. chesapeaki* was low in the 2000s [8] but then experienced a sharp increase, as reflected in a 2014 PCR survey [10]. Several hypotheses could help to explain the increase in PCR-prevalence and range extension of these parasites. These include: (1) Introduction/evolution of strains of the parasite adapted to host and/or environmental conditions in Maine, (2) Propagation of more susceptible oyster hosts that favor disease transmission by aquaculture activities and (3) The overall increase in water temperatures in the Gulf of Maine [20]. In this study, we expanded our 2014 parasite survey of the eastern oyster [10] via qPCR (quantitative polymerase chain reaction) detection of *H. nelsoni*, *P. marinus,* and *P. chesapeaki* over two-years in summer and early fall at six sites in Maine (Figure 1).

## 2. Results

### 2.1. Haplosporidium nelsoni (MSX)

**Figure 2:**
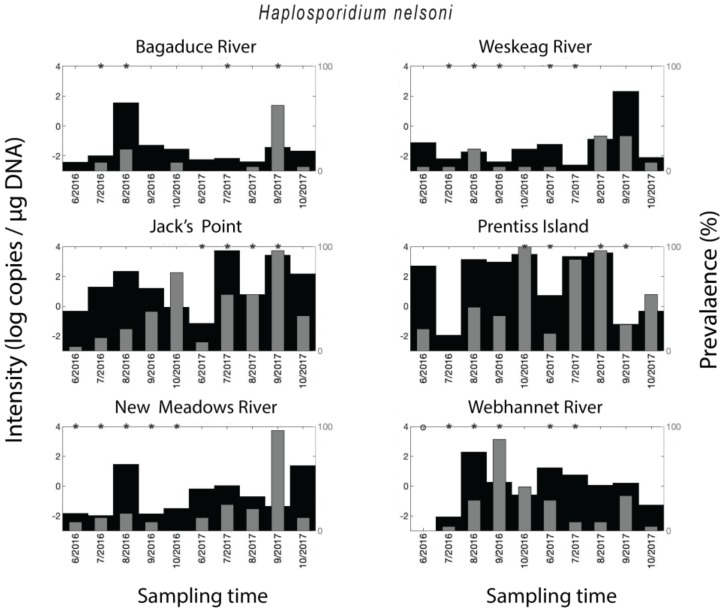
Spatial–temporal patterns for *Haplosporidium nelsoni*. Parasite density and parasite qPCR-prevalence. * indicates a statistically significant (*p* < 0.05) difference in parasite density when comparing two neighboring time points.

*Crassostrea virginica* samples tested positive for *H. nelsoni* in 62% of all qPCR assays. The qPCR-prevalence of *H. nelsoni* was higher in 2017 (74%) than in 2016 (50%), except for the Webhannet River, where the trend was opposite, with higher qPCR-prevalence in 2016 compared to 2017. The year-round qPCR-prevalence for MSX was medium-high for Jack’s Point (40%) and Prentiss Island (48%) in the DRE (Damariscotta River Estuary), and Webhannet River (25%), low for Bagaduce River (11%) and Weskeag River (12%), and medium for New Meadows River (21%). A seasonal trend in *H. nelsoni* qPCR-prevalence was observed at all of the sites for both years, where qPCR-prevalence of infection increased over the course of the sampling season to a peak value followed by an immediate decrease (Figure 2). The timing of the peaks in qPCR-prevalence was location-dependent. Still, in all the samples with medium (New Meadows River) to medium-high qPCR-prevalence (Jack’s Point and Prentiss Island, and Webhannet River), it occurred in the fall (Figure 1). In some sites/times, the parasite density (copies per µg DNA) mimicked the qPCR-prevalence data and was higher for the sites/years with the highest qPCR-prevalence. However, overall, the qPCR-prevalence and parasite density varied independently (Figure 2).

### 2.2. Perkinsus spp. (Dermo)

**Figure 3:**
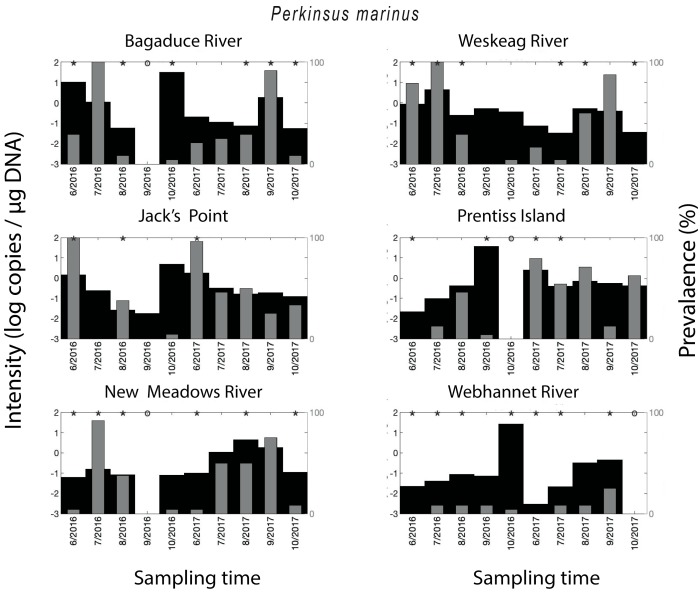
Spatial–temporal patterns for *Perkinsus marinus*. Parasite density and parasite qPCR-prevalence. * indicates a statistically significant (*p* < 0.05) difference in parasite density when comparing two neighboring time points.

*Crassostrea virginica* tested positive for *P. marinus* in 57% of all qPCR assays. Compared on a year to year basis, the qPCR-prevalence of *P. marinus* in 2016 (45%) was substantially lower compared to its qPCR-prevalence in 2017 (68%) (Figure 3). The year-round mean qPCR-prevalence for *P. marinus* ranged from 7–39% depending on the site—Bagaduce River (32%), Weskeag River (37%), Jack’s Point (39%), Prentiss Island (34%), New Meadows River (33%), Webhannet River (7%). The seasonal trend for *P. marinus* qPCR-prevalence varied between sites, with peaks at various times throughout the summer, but in some cases, there was no clearly discernible peak (Figure 2). As with MSX, the parasite density of *P. marinus* mimicked its trend in qPCR-prevalence and was higher for the sites/years with the highest qPCR-prevalence. We also observed years with low qPCR-prevalence and high parasite density (Figure 3).

**Figure 4:**
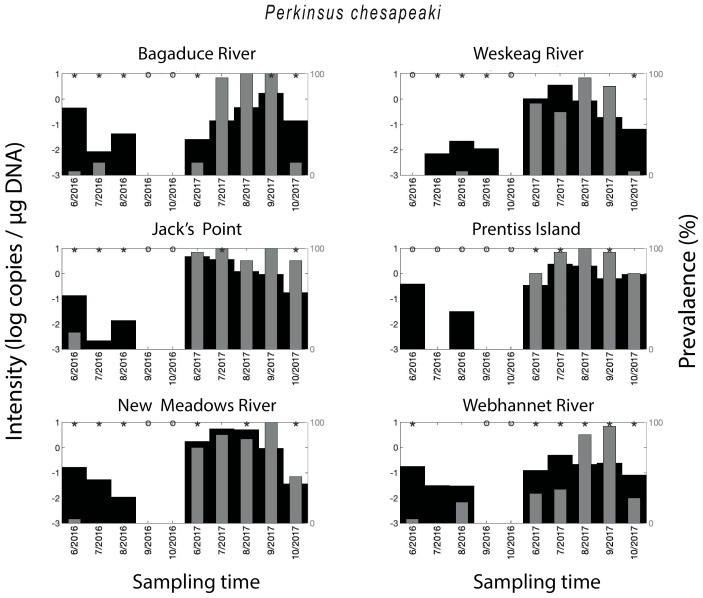
Spatial–temporal patterns for *Perkinsus chesapeaki*. Parasite density and parasite qPCR-prevalence. * indicates a statistically significant (*p* < 0.05) difference in parasite density when comparing two neighboring time points.

**Figure 5:**
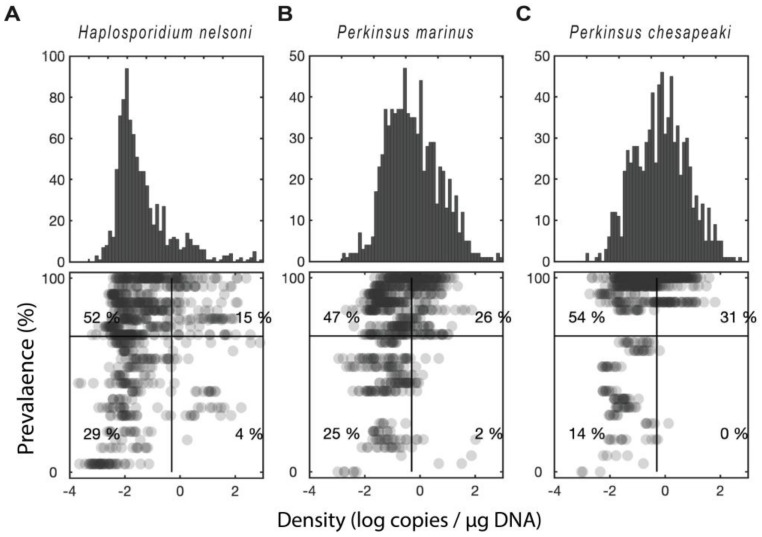
Distribution of prevalence versus intensity for the sampled sites. (**A**) *Haplosporidium nelsoni* (**B**) *Perkinsus marinus*. (**C**) *Perkinsus chesapeaki*. Upper panel shows a histogram of parasite density (log scale). Lower panel shows a scatter plot of infection intensity versus infection prevalence (percentage of oysters infected at a given site/time). The numbers indicate the percentage of points that fall within each quadrant.

*Perkinsus chesapeaki* was detected in all of the sampled areas, with 57% of all oysters testing positive for the parasite. The qPCR-prevalence of *P. chesapeaki* in 2017 (96%) was much higher than in 2016 (18%), with samples from some months, across all sites, showing no detection of the parasite at all. This was especially true for oysters from Weskeag River, Jack’s Point, Prentiss Island, and New Meadows River (Figure 4). The two-year mean qPCR-prevalence for *P. marinus* was particularly high for Jack’s Point (94%) and Prentiss Island (88%), and also above 50% for Bagaduce River (64%), Weskeag River (64%), New Meadows River (78%), and Webhannet River (64%). A seasonal trend in *P. chesapeaki* qPCR-prevalence was observed in 2017 with a peak in the summer months (Figure 5). With some exceptions, the parasite density mimicked the qPCR-prevalence and was higher in the months with the highest qPCR-prevalence (Figure 4).

### 2.3. Distribution of qPCR-Prevalence Versus Density for the Sampled Sites

When comparing qPCR-prevalence versus parasite density within each parasite, *P. marinus* and *H. nelsoni* had a similar level of correlation (r^2^ = 0.09, *p* < 0.001 for both, Figure 5a,b). *P. chesapeaki* had the highest correlation (r^2^ = 0.16, *p* < 0.001, Figure 5c). These correlations show a high level of significance but low r^2^ because there is a substantial deviation from a linear relationship. For example, approximately half of the oysters (56%) fell into either the high-density-low-qPCR-prevalence or the low-density-high-qPCR-prevalence quadrants—a pattern that was counter to the correlation. Across all three parasites, the most common scenario by far was a low parasite density, but high qPCR-prevalence. Thus, many of the oysters in our study had low levels of infection by one or more of the parasites, while a smaller proportion of the oysters had high levels of infection.

### 2.4. Statistical Analysis

When viewing risk ratios, the presence of one parasite influenced the presence of a second parasite with 95% confidence in all cases. The presence of *H. nelsoni*, *P. marinus*, and *P. chesapeaki* all increased the risk of having a second parasite, though only slightly (Table 1). At a significance level of *p* < 0.01, *P. chesapeaki* correlated with *P. marinus* and *H. nelsoni*, (*r*^2^ = 0.12, 0.06 respectively). All parasites were positively correlated with each other (*p* < 0.05). Analysis of temperature and salinity data did not identify consistent drivers of infection for the three parasites in the current study.

## 3. Discussion

There are multiple ways to detect protozoan parasites in bivalves, with PCR being often the most rapid, sensitive, and specific of the techniques [21,22,23], not to mention that these techniques are primed for high throughput and multiplex surveys [8,24,25,26,27]. Furthermore, more groups continue to develop DNA-based detection techniques [28,29] and use these to identify new host species [30,31]. We also stand with the argument expressed in a previous paper that the identification of a specific parasite in the type of host is a proxy for infection/colonization independently of whether the oyster manifests signs of the disease [7]. In brief, the parasite adherence to the host is part of the infection cycle [32,33,34,35,36] since the parasite changes the gene expression once it reaches the pallial cavity and translocates throughout the host’s soft body [37,38]. We should also note that to date, all the *Perkinsus* spp. have been described as parasites of mollusks and they have evolved to adhere to, enter the oyster, and survive inside the defense cells of mollusks [35,36,39] (reviewed in [40]). Consequently, the protozoan detected in the oysters can only come from infected oysters. When a parasite is described for the first time in a new host, it is important to use, when possible, multiple techniques to define the nature of the interaction (e.g., in [41]). Equally important for the choice of the diagnostic technique is to know the limits of the technique and the objective of the study; e.g., Ray’s fluid thioglycollate medium technique (RFTM) provides a robust way to quantify parasite cells in the bivalve soft-tissues (reviewed in [42]). Our study was intended to establish a base-line for the presence of the studied protozoans using DNA-based assays. Other techniques (e.g., histopathology, RT-PCR (reverse transcriptase-PCR)) would have added information about the dynamic of the infection and the reaction of the host; however, pursuing these objectives was outside the scope of this study.

### 3.1. Haplosporidium nelsoni qPCR-Prevalence and Distribution

*Haplosporidium nelsoni* was first reported in Maine waters from oysters that were sampled from the Piscataqua River, which defines the border between the states of Maine and New Hampshire, and first showed up with a PCR-prevalence ranging from 15% to 81% [13]. *H. nelsoni* kept a low profile in Maine, until the oyster die-off reported in 2010 [14,15]. In response to this die-off, the oyster aquaculture industry switched to a strain of oyster that was resistant to MSX infections [16]. The reduction in the PCR-prevalence of MSX in commercially harvested oysters compared to previous surveys has been interpreted as evidence that the threat from MSX has been neutralized [17]. Nevertheless, results from three independent groups indicate low/medium PCR-prevalence for *H. nelsoni* in oysters from Maine during 2013–2014 [9,10,11,12,18]. The PCR-prevalence detected in the latest surveys performed in the DRE (2014 and two sites, one sample in 2016) was variable depending on sampling location (3–53%), with the highest PCR-prevalence in the summer/fall months [12]. Our survey included six commercial sites during the years 2016 and 2017 with no exact overlap with the survey conducted by Dickey et al. (2017) [12]; the closest points were Prentiss Island and Jack’s Point, also in the DRE. The qPCR-prevalence of MSX at these two sites was the highest within sites included in the current study and also higher than that reported previously for the DRE [9,10,11,12,18]. In total, the data from all recent studies suggest that the parasite is not only present but also able to reach high prevalence and density in oysters. Unfortunately, whether the oysters sampled in the present study were the MSX-resistant strain remains an open question as we are not aware of any genetic marker to identify them as such.

Smaller bodies of water with less oyster aquaculture activity (New Meadows and Webhannet Rivers) also reached punctuated, high qPCR-prevalence during some months of the year. Over the 2-year survey, we observed high variability in the qPCR-prevalence of MSX with a combination of high and low levels of infection. The use of MSX resistant strains may be the reason that die-offs were not observed at locations with high MSX qPCR-prevalence and density; however, the assessment of oyster mortality was outside the scope of the study, and no oyster die-offs were brought to our attention during the survey. Nevertheless, our findings strongly suggest that oysters are still becoming infected with *H. nelsoni* and in some places/dates at very high qPCR-prevalence. This is particularly true for the oysters grown in the DRE, which contains 85–90% of the oyster production in Maine. It is interesting to note the relatively high parasite density in months where qPCR-prevalence values were low. At the same time, across all three parasites, the most common scenario was a low parasite density, but high qPCR-prevalence. At this point, we cannot explain why high qPCR-prevalence has not resulted in mass mortalities. One hypothesis to explain this result would be that mortalities are happening, but dead individuals are not sampled. We relied on samples provided by the oyster farmers, and the study did not include an assessment of the overall mortality of oysters at our study sites. However, during the current survey, large-scale die-offs were not reported by oyster farmers or marine resource managers. Although much is known about the distribution and qPCR-prevalence of *H. nelsoni* in oysters, its life cycle remains to be ascertained [43] and to our knowledge, no clear mechanism of resistance against MSX has been identified [16]. Transmission of *H. nelsoni* between oysters has been shown [44], and the tunicate *Styela* sp. has been hypothesized as a reservoir [11]. Recently, *H. nelsoni* has been reported as causing systemic infection in *Crassostrea gigas* from Ireland [45]. The report identifies plasmodia of the parasite found within the connective tissue of the oyster’s digestive gland, and spores attributed to the parasite within the lumen of the intestine [45]. Our qPCR-based survey revealed medium to high qPCR-prevalence of *H. nelsoni* in farmed oysters for most of the sampled areas, confirming the broader distribution of *H. nelsoni* as suggested by previous studies [9,10,12]. Management of oyster resources around MSX is quite challenging, given the unknown aspects of the *H. nelsoni* life cycle. The present qPCR survey lacks histopathology of parasites in oyster tissues and information regarding the genotype of the sampled oysters (diploid vs. triploid, oyster strains origin), which adds a layer of complexity to interpreting the high qPCR-prevalence in the absence of reported die-offs. Finally, the contribution of wild oyster populations to the parasite load in the water column is unknown. This is due to the acknowledged gaps in the *H. nelsoni* life cycle, but also because any die-offs affecting wild oyster populations are likely to pass unnoticed because most mollusk pathogens are not associated with any gross signs of disease [46].

### 3.2. Perkinsus spp. qPCR-Prevalence and Distribution

The protozoan parasites *P. marinus* and *P. chesapeaki* are two of the six species within the genus *Perkinsus*, and both of them have been reported in oysters from Maine [8,10,47]. Since the first report of *P. marinus* in Maine [19] there have been 11 additional references to *Perkinsus* spp. [7] with the latest report using qPCR-based assays indicating a 15-65-fold increase of the *P. marinus* and *P. chesapeaki* qPCR-prevalence over 12 years [10]. In this study, we sampled four of the sites reported in the study by Marquis et al. (2015), including the Bagaduce River, the Webhannet River, the Weskeag River, and the New Meadows River. Jack’s Point and Prentiss Island were located North of the Jones Cove site sampled by Marquis et al. (2015). We detected *Perkinsus* spp. at each of the six sites that were sampled in the current study. The qPCR-prevalence of *P. marinus* varied between years, with 2017 having a higher qPCR-prevalence. In the case of *P. chesapeaki*, it was notable that its near absence during 2016 was followed by a very high qPCR-prevalence in 2017, reaching 100% of the individuals on several dates and from several locations. *P. chesapeaki* was initially described from infected clams, and in the past, it was thought that *P. chesapeaki* had a preference for infecting clams [47,48]. A shellfish survey conducted in 2002, for both oysters and clams from the East Coast of the USA, also supported the notion that *P. chesapeaki* preferentially infected clams with the highest qPCR-prevalence of *P. chesapeaki* in oysters at 6.9% in Walpole, Maine, from a site located in the DRE [8]. However, oysters from Jones Cove, just 7 miles south of Walpole, showed a qPCR-prevalence of *P. chesapeaki* reaching values of 43.5% in 2014 [10]. The high qPCR-prevalence of *P. chesapeaki* can be interpreted in several ways. The parasite may have adapted to infect the oyster strains growing in Maine’s cold waters or to the new and resistant aquaculture strain of *C. virginica*. Alternatively, the translocation of *P. chesapeaki* strains from other sites may explain the higher qPCR-prevalence values. Translocation of *P. marinus* between the Gulf of Mexico and the Pacific Ocean has been described based on genetic studies [49]. Still without an exhaustive genetic analysis of *P. marinus* and *P. chesapeaki* isolated from Maine oysters, translocation cannot be ruled out as an explanation for recently higher qPCR-prevalence values in Maine. Interestingly, oyster strains tested in Maine experienced higher mortalities compared to other sites, and the cause was attributed to the time the oyster seeds were deployed [16]; in the same study (2012–2013), the “Dermo pressure” was rated as low in the DRE using qPCR that did not include *P. chesapeaki*. This study was carried out right before our first large survey in Maine (2014), where we reported the 65-fold increase of *P. chesapeaki* PCR-prevalence over 12 years [10].

### 3.3. Parasite Density

In our 2015 survey, we used endpoint qPCR assays to diagnose the presence of oyster pathogens [21,25,50]. Consequently, the samples were scored as positive or negative for each of the parasites and sites. Here, we used qPCR assays [21,23,50,51,52] to estimate gene copy number and provide a proxy for the parasite density. The rRNA gene cluster in the *Perkinsus* spp. studied is arranged in head to tail copies [24,50,53,54] and for *P. chesapeaki* it is estimated to be present in the range of 500 copies/cell [54]. The nature of the targeted genes, together with the propagation by schizogony, a process where a single trophozoite can generate up to 64 daughter cells [55], would help to explain the high copy number observed in some samples. To our knowledge, there is no similar data for the rRNA gene organization in the *H. nelsoni* genome. For both parasite genera, it is not currently possible to transform qPCR-based copy numbers into probable cell numbers due to complex life cycles, propagation strategies, and for *H. nelsoni*, its multiple nuclei. Nevertheless, the copy number/µg of extracted DNA provided a proxy for the parasite density. The parasite density reached a peak of almost 10,000 copies/µg of DNA in July 2017 for *H. nelsoni*. Studies using RFTM in clams have reported values of up to 3.9 × 10^6^ cells /individual [56] and 10^6^ cells/g wet tissue [57]. Futures studies targeting some of the sites in this survey could incorporate such techniques as the RFTM to provide a total cell number for the individuals sampled.

### 3.4. Parasite Association

Clams (*Tapes semidecussatus*) heavily infected with *P. atlanticus* were co-infected by bacteria and/or viruses compared to neither noninfected or lightly *P. atlanticus*-infected specimens [58]. We have reported in the past that oysters positive for both *Perkinsus* spp. are 3.2 times as likely to be positive for a non-*Perkinsus* protozoan parasite [10]. We analyzed the association between oyster parasites, including recent data for the protozoan parasites of humans *Toxoplasma gondii* and *Cryptosporidium parvum* found in oysters [59]. In the present study, we found a moderately increased (and statistically significant) risk association for oysters infected with *Perkinsus* spp. The smaller risk ratio maybe because the present study covers multiple seasons and years, including a broader range of conditions. The two human parasites also fell into a bimodal distribution of parasite density [59], a pattern that was not observed for any of the oyster parasites, which all fell into a unimodal distribution. This could represent a difference between parasites that interact dynamically with the oyster immune system and those that simply pass through so that parasite density simply reflects the relative abundance of parasites in surrounding waters. Interestingly, the micro-eukaryotic diversity in the oyster aquaculture areas (2 liter water samples) showed no presence of the specific parasites in the current study, suggesting either a rapid dispersion or a rapid uptake by the hosts (P.D. Countway, N.D. Marquis, N.R. Record, J.A. Fernández Robledo, unpublished work).

## 4. Materials and Methods

### 4.1. Oyster Sampling and DNA Extraction

*Crassostrea virginica* specimens (n = 24 oysters from each sampling site, Supplementary Table S1) were collected monthly from June through October during 2016 and 2017, at shellfish leases along the Maine coast: Bagaduce River (Brooksville), Weskeag River (Thomaston), Prentiss Island (Bristol) and Jack’s Point (Newcastle) in the DRE, New Meadows River (Bath), and Webhannet River (Wells) (Figure 1). Samples were delivered on land or to the laboratory in coolers with pocket ice packs by the oyster growers; the samples were maintained in refrigeration and time between the collection and the dissection varied between a few hours up to two days.

Oysters were dissected, and tissue subsamples (rectum, gill, and mantle) were collected from each individual and pooled (50–100 mg wet weight of total tissue/pool). DNA was extracted from pooled tissue samples using a commercial kit (Omega Biotek E.Z.N.A. Tissue Kit, Norcross, GA, USA). DNA concentration and purity were estimated with a Nanodrop^TM^ 2000 spectrophotometer (ThermoFisher Scientific, Grand Island, NY, USA). To facilitate the manual high throughput and to avoid individual volume adjustments, DNA samples were diluted and aliquoted to contain 200–600 ng of DNA [8,10,47] in a volume of 3 µL. A few samples required further adjustment by concentrating the DNA. All aliquoted samples were stored at −20 °C until tested by qPCR for each of the protozoan parasites. After the qPCR results, the copy number was standardized to copies in 1 µg of DNA.

### 4.2. qPCR Diagnostic Tests

**Figure 6:**
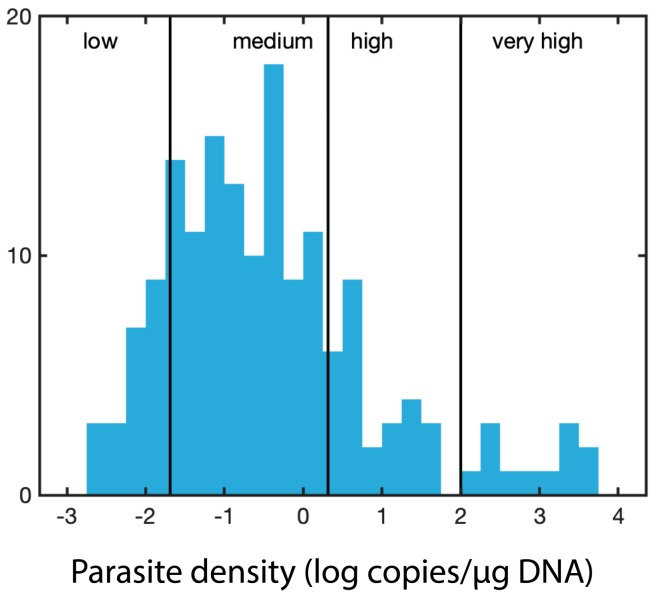
Cutoffs for the parasite density based on the distribution of the parasite density values (log-normally distributed).

For each sample, parasite-specific qPCR assays were run for *H. nelsoni* (forward primer 5′- GGG CTA ATA CGT GAT AAA TGG TAC G -3′; reverse primer 5′- GAT TCC CCG TTA CCC GTC AT -3′), which target the small subunit of the rRNA gene [23], *P. marinus* (forward primer 5′- CAC TTG TAT TGT GAA GCA CCC - 3′; reverse primer 5′- TTG GTG ACA TCT CCA AAT GAC -3′) [21,51], *P. chesapeaki* (forward primer 5′- AAG TCG AAT TGG AGG CGT GGT GAC -3′; reverse primer 5′- ATT GTG TAA CCA CCC CAG GC -3′) [50,52]. The sets of primers for *P. marinus* and *P. chesapeaki* target the NTS (nontranscribed spacer) that separates the rRNA gene clusters [21,50,51,52]. Primers and probes were evaluated using the IDT Primer Quest tool and AlleleID^®^ v.7 (PREMIER Biosoft, Palo Alto, CA, USA), purchased from Integrated DNA Technologies (IDT Inc., Coralville, IA, USA). The labeled probes were 5′- /6FAM/ ACA AAT CAT TCA AGT TTC TGC /MGBNFQ/ -3′, 5′-/ TexRd-XN/ ACA TGG GCG AAA TTG ACT TGC AGG /IAbRQSp/ -3′, and 5′- HEX/TTT GGC CGG/ZEN/TTC ATT GGT GTC AAA/3IABKFQ/ -3′ for *H. nelsoni*, *P. marinus*, and *P. chesapeaki*, respectively. The probes utilized dual-quenching with ZEN™ and Iowa Black^®^ (IDT Inc., Coralville, IA, USA) to decrease background fluorescence. The qPCR assays were performed on a Bio-Rad CFX96 optical unit equipped with a C1000 thermal cycler (Bio-Rad, Hercules, CA, USA). Bio-Rad skirted and semi-skirted 96 well plates were sealed with Microseal ‘B’ optical film (Bio-Rad). The total reaction volume (25 µL) consisted of 12.5 µL of PrimeTime^®^ Gene Expression Master Mix (IDT), 0.5 µL of 10 µM probe, and 0.5 µL each of 10 µM forward and reverse primers. The qPCR reaction was completed with 8 µL of water and 3 µL of extracted DNA. DNA standards for qPCR were prepared from qPCR-positive samples that were cloned, plasmid purified, linearized with *Not*I, and diluted over five orders of magnitude (ranging from 10^6^ to 10^1^ copies per reaction). All qPCR included an initial denaturation step at 95 °C for 3.0 min and a total of 35 cycles. The qPCR conditions for the *H. nelsoni* were 95 °C for 30 sec, 60 °C for 30 sec, and 72 °C for 30 sec. For *P. marinus* 95 °C for 20 sec and 57 °C for 50 sec and for *P. chesapeaki* 94 °C for 25 sec and 59.5 °C for 50 sec. Exact copy numbers per microliter of the undiluted DNA standards were determined from the DNA concentration of linearized plasmid, and the exact molecular weight of each cloned qPCR product plus the vector. Exact copy numbers for a particular dilution series varied slightly depending on the batch of a particular linearized standard that was used over the two-year project. All samples and standards were run in triplicate to assess the variation of technical replicates. ‘Parasite density’ was defined simply as gene abundance/μg DNA. The distribution of the parasite density values was log-normally distributed, with an additional high tail to the right (Figure 6). We divided the log-normal portion of the distribution (<100 copies per µg) into three rankings: low (lowest 12.5%, < 0.02 copies/µg DNA, determined as less than one standard deviation from the mean), medium (up to 70%, between 0.02 and 2 copies/µg DNA, within one standard deviation from the mean), and high (up to 83%, between 2 and 100 copies/µg DNA). We included a fourth ranking for the high tail to the right (with measurements of >100 copies/µg DNA), which we ranked as very high parasite density.

### 4.3. Environmental Data

**Figure 7:**
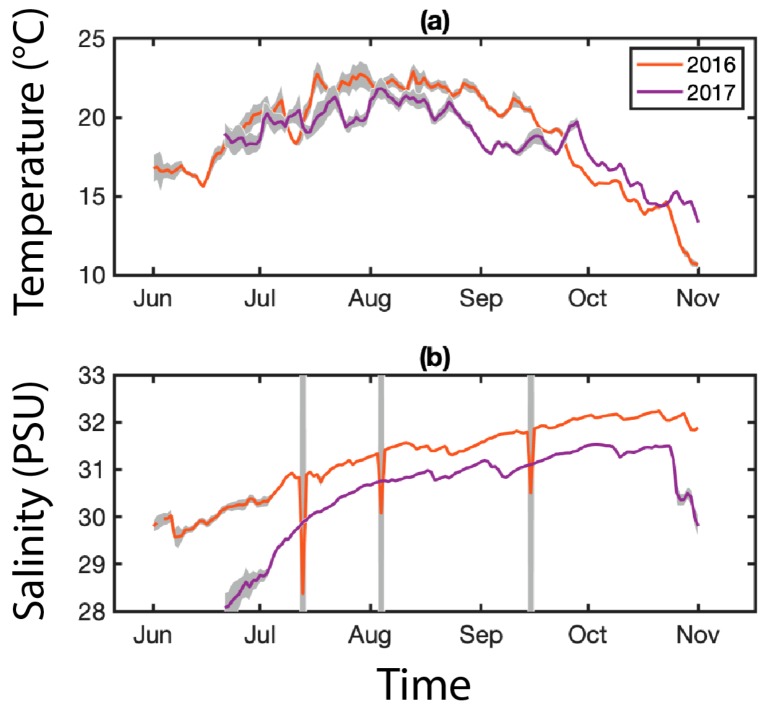
Environmental parameters for the Damariscotta River Estuary (DRE). An array of LOBO buoys are deployed along the coast of Maine as part of the University of Maine’s SEANET project. One of these buoys was located near the Prentiss Island site from 2015–2018, collecting hourly temperature (°C, (**a**)) and salinity (PSU, (**b**)) data. Data are averaged daily; gray shading indicates daily variance.

For the environmental temperature (°C) and salinity (PSU), we relied on the array of Land/Ocean Biogeochemical Observatories (LOBO) buoys (University of Maine’s SEANET project) deployed near the Prentiss Island site in the DRE (Figure 7). We used these data to examine possible environmental controls on the prevalence and density of MSX and Dermo infections. For comparison with parasite data, environmental data were averaged to monthly means corresponding to oyster sample months. Basic exploratory statistical analyses were performed, including Pearson linear correlation coefficients and two-sample t-tests. No buoys were deployed near the other sample sites.

### 4.4. Statistical Analysis

The main focus of this study was to assess and compare infection frequencies of *H. nelsoni* and *Perkinsus* spp. collected from oysters at several sampling sites for over 2 years. Statistical analyses were performed using Matlab (MathWorks, Inc., Natick, MA, USA). Changes in parasite density over time were labeled statistically significant if the mean measured copies/µg DNA at a point in time was different from the mean measured at neighboring time points (*p* < 0.05, two-sample t-test), with the understanding that this labeling was primarily for descriptive purposes rather than formal statistical hypothesis testing. To address the question of whether infection by one parasite makes an oyster more susceptible to infection by another parasite, we calculated the risk ratio for each parasite per sample, where ‘exposure’ was defined as infection by a given parasite, and the ‘outcome’ was defined as infection by another parasite, so that the risk ratio gives the relative increase in infection risk for a given parasite when another parasite is present. For oysters positive for the parasites, we also examined pairwise correlations of parasite intensities (log copies/µg DNA).

## 5. Conclusions

The qPCR-based survey indicates that *P. marinus*, *P. chesapeaki*, and *H. nelsoni* are present in all the six sampled sites from Maine. Consequently, Maine provides the environmental and biological conditions (natural oyster beds and aquacultured oysters) to propagate and transmit the pathogens between oysters. Interestingly, during the study period (2016–2017) and until now (winter of 2019) no die-offs have been reported, indicating that the epizootiological triad integrated by protozoans, host fitness, and environmental conditions are enough to sustain the propagation the parasites with no noticeable mortalities. These protozoan parasites are in Maine to stay.

## Figures and Tables

**Table 1:** Risk ratios for each parasite, given exposure to another parasite, plus 95% confidence intervals.

		Outcome Parasite		
		*P. chesapeaki*	*P. marinus*	*H. nelsoni*
**Exposure parasite**	***P. chesapeaki***		1.8 ± 0.06	1.43 ± 0.05
	***P. marinus***	1.78 ± 0.05		1.14 ± 0.04
	***H. nelsoni***	1.51 ± 0.05	1.16 ± 0.05	

## Supplementary Materials

The following are available online at https://www.mdpi.com/2076-0817/9/4/256/s1, Table S1: Biometrics of the sampled oysters (n=24 for each sampling site) during the study.
