# Supplementary material for: A qPCR-Based Survey of Haplosporidium nelsoni and Perkinsus spp. in the Eastern Oyster, Crassostrea virginica in Maine, USA
Source: Pathogens. 2020 Mar 31;9(4):256. doi: 10.3390/pathogens9040256 (PMC7238206; doi:10.3390/pathogens9040256)
Supplement: Supplementary file 1 [file pathogens-09-00256-s001.pdf]

Supplementary Table 1. Biometrics of the sampled oysters (n=24 for each sampling site) during the study.

		2016				2017			
Location	Month	Weight (g)	±SD	Length (mm)	±SD	Weight (g)	±SD	Length (mm)	±SD
Bagaduce River	Jun	39.33	6.38	60.21	3.24	39.54	6.09	61.73	4.08
	Jul	37.08	5.00	71.88	2.47	45.21	5.42	77.46	4.22
	Aug	42.71	6.92	76.79	4.54	54.88	8.65	80.92	4.94
	Sept	56.00	8.24	84.46	6.81	63.25	6.40	89.50	5.82
	Oct	50.88	6.29	79.08	3.39	65.12	6.87	82.67	4.90
Weskeag River	Jun	75.04	21.38	85.13	6.54	66.96	14.21	87.10	11.98
	Jul	43.67	7.29	60.00	6.60	92.00	23.11	92.96	10.08
	Aug	66.29	22.02	84.42	13.49	93.64	49.77	87.62	17.95
	Sept	54.75	10.6	76.92	5.60	133.38	54.79	103.12	15.36
	Oct	94.17	33.84	92.50	17.50	123.04	47.61	105.71	16.36
Jack’s Point	Jun	34.96	6.22	74.5	6.82	38.5	9.41	57.42	5.06
	Jul	26.08	4.35	65.5	5.07	43.29	8.18	76.42	4.99
	Aug	47.75	5.33	77.62	5.05	50.54	11.08	80.29	7.18
	Sept	27.62	4.92	73.38	6.21	41.96	9.76	83.08	10.59
	Oct	64.08	9.04	85.5	7.34	40.67	8.67	81.21	6.21
Prentiss Island	Jun	66.75	21.45	87.74	10.77	53.29	9.89	75.16	6.68
	Jul	69.67	15.24	89.75	10.49	66.92	19.94	88.33	8.88
	Aug	67.79	11.92	92.21	9.05	68.04	14.99	90.00	10.58
	Sept	83.92	16.2	94.58	13.29	63.00	13.56	98.29	7.74
	Oct	74.54	12.23	90.04	8.66	42.29	7.12	85.12	8.37
New Meadows River	Jun	86.46	15.16	83.75	9.24	40.79	16.18	64.37	5.68
	Jul	86.46	12.05	103.00	9.86	50.33	10.61	84.88	8.01
	Aug	97.62	15.72	103.96	11.72	55.25	11.82	87.83	10.09
	Sept	44.33	10.26	78.50	6.28	48.62	8.67	84.33	6.67
	Oct	38.33	6.99	82.04	8.62	45.71	10.40	86.62	8.43
Webhannet River	Jun	47.96	9.63	86.50	8.18	67.54	18.80	74.55	12.11
	Jul	52.42	13.07	68.08	8.39	58.00	13.02	79.04	10.1
	Aug	47.42	8.45	76.79	6.55	58.00	13.02	79.04	10.1
	Sept	46.46	10.37	79.92	6.73	103.54	32.72	99.08	15.57
	Oct	83.42	19.03	99.08	14.23	58.58	12.20	76.25	8.57
